# Multidisciplinary Management of Cutaneous Squamous Cell Carcinoma of the Scalp: An Algorithm for Reconstruction and Treatment

**DOI:** 10.3390/jcm13061581

**Published:** 2024-03-10

**Authors:** Manuela Rodio, Matilde Tettamanzi, Emilio Trignano, Silvia Rampazzo, Pietro Luciano Serra, Federica Grieco, Riccardo Boccaletti, Filippo Veneziani Santonio, Giovanni Maria Fadda, Fabrizio Sanna, Dalila Di Mario, Corrado Rubino

**Affiliations:** 1 Plastic Surgery Unit, University Hospital Trust of Sassari, 07100 Sassari, Italy; m.tettamanzi@studenti.uniss.it (M.T.); emilio.trignano@aouss.it (E.T.); s.rampazzo@studenti.uniss.it (S.R.); p.serra4@studenti.uniss.it (P.L.S.); f.grieco1@studenti.uniss.it (F.G.); corubino@uniss.it (C.R.); 2 Plastic, Reconstructive and Aesthetic Surgery Training Program, University of Sassari, 07100 Sassari, Italy; 3 Department of Medicine, Surgery and Pharmacy, University of Sassari, 07100 Sassari, Italy; 4 Department of Neurosurgery, Azienda Ospedaliero Universitaria di Sassari, 07100 Sassari, Italy; riccardo.boccaletti@aouss.it (R.B.); filippovenezianis@gmail.com (F.V.S.); 5 Unità Operativa Complessa di Oncologia Medica, Azienda Ospedaliera Universitaria di Sassari, 07100 Sassari, Italy; giovanni.fadda@aouss.it; 6 Radiotherapy Department, Sassari Hospital, University of Sassari, 07100 Sassari, Italy; fabriziosannasassari@yahoo.it (F.S.); dalila.dm@tiscali.it (D.D.M.)

**Keywords:** cutaneous squamous cell carcinoma (cSCC), scalp, multidisciplinary, algorithm, reconstruction, radiotherapy, immunotherapy, cemiplimab

## Abstract

**Background**: Scalp-associated cutaneous squamous cell carcinoma (cSCC) presents formidable treatment challenges, especially when it leads to full-thickness defects involving bone. Aggressive or recurring cases often demand a multidisciplinary approach. Leveraging our surgical experience and a literature review, we introduce a therapeutic algorithm to guide the selection of reconstruction methods, particularly for locally advanced lesions, furthermore showing the synergy between surgery and other therapies for comprehensive, multidisciplinary disease management. **Methods**: Our algorithm stems from a retrospective analysis of 202 patients undergoing scalp cSCC resection and reconstruction over a 7-year period, encompassing 243 malignancies. After rigorous risk assessment and documentation of surgical procedures, reconstruction methods were therefore related to malignancy extent, depth, and individual clinical status. **Results**: The documented reconstructions included 76 primary closures, 115 skin grafts, 7 dermal substitute reconstructions, 33 local flaps, 1 locoregional flap, and 1 microsurgical free flap. Patients unsuitable for surgery received radiotherapy or immunotherapy after histological confirmation. Precise analysis of tumor characteristics in terms of infiltration extent and depth guided the selection of appropriate reconstruction and treatment strategies Combining these insights with an extensive literature review enabled us to formulate our algorithm for managing scalp cSCCs. **Conclusions**: Effectively addressing scalp cSCC, especially in locally advanced or recurrent cases, demands a systematic approach integrating surgery, radiotherapy, and immunotherapy. Our multidisciplinary team’s decision-making algorithm improved patient outcomes by offering a broader spectrum of therapeutic options that can synergistically achieve optimal results.

## 1. Introduction

Cutaneous squamous cell carcinoma (cSCC) represents the second most common type of non-melanoma skin cancer (NMSC), preceded in frequency only by basal cell carcinoma. Caucasian individuals are more often affected, with an average age of onset after the mid-sixth decade of life and an estimated lifetime incidence of about 7–11% in the U.S.A. The disease has a predilection for males [1]. Many risk factors have been described, such as ionizing radiation, chronic immunosuppression, HPV infections, and previous burns, but above all, exposure to ultraviolet radiation and sunlight has a direct correlation with the incidence of SCC [1]. This explains the greater onset of lesions in photo-exposed body regions, mainly the head, neck, and face, and with advancing age. Among the photo-exposed areas, the scalp is one of the most frequently affected by cutaneous malignancies, and almost 16% of these are squamous cell carcinomas [2].

Scalp tissue losses following the resection of skin malignancies can result in complex wounds, which may represent a challenge for plastic surgeons regarding reconstruction, especially in extensive and full-thickness defects involving periosteum and even bone and dura mater [3]. The difficulties placed by the treatment and reconstruction of large defects of the scalp are many: the scalp has intrinsic characteristics that sometimes restrict reconstructive options, such as limited tissue movement due to scalp thickness and the presence of inelastic galea aponeurotica, a paucity of adjacent donor tissues, limited tissue expandability, and the convexity of the cranium surface; another problem is represented by the necessity of the replacement of hair-bearing tissue in some cases to achieve an adequate aesthetic appearance; moreover, scalp tumors are often wide and locally advanced at the moment of clinical presentation, and when there is calvarial bony and dura involvement, a complex surgical program needs to be set with the attendance of a neurosurgical team [3,4]. Many reconstructive alternatives have been proposed in the literature according to the features of tissue losses, from the simplest ones, like primary closure for small defects, local flaps set up from adjacent scalp skin for small–medium-sized defects, and skin grafting (partial- or full-thickness) when calvaria periosteum is preserved, to the more complex ones, like reconstruction with dermal substitutes and subsequent skin grafting, locoregional flaps, and microsurgical free flaps for full-thickness scalp tissue losses involving the calvaria. The issue is choosing the best reconstructive option considering the patients’ functional and aesthetic needs. From this perspective, preoperative planning acquires even more importance regarding the study of associated comorbidities, which may sometimes limit anesthetic tolerance and complex reconstructions [4]. Management of the most aggressive or recurrent lesions often requires a multidisciplinary approach.

Most cases of cSCC are associated with a favorable outcome and undergo resolution with radical surgery and proper reconstruction, but patients with high-risk features present with a higher risk of local recurrence and nodal metastasis; for this reason, it is crucial to include an accurate definition of “high-risk” lesions with the aim of prognosis definition and treatment planning [5].

The management of squamous cell carcinoma of the scalp, especially in locally advanced and recurrent cases, requires a systematic approach to the disease, combining surgery with radiation therapy, chemotherapy, and immunotherapy. Patients with advanced cSCC are often deemed ineligible for either or both of curative surgery and radiation therapy and, until recently, were limited to off-label systemic cisplatin–fluorouracil or cetuximab therapy, which offers modest clinical benefits and potentially severe toxicity. A new systemic therapy, Cemiplimab, has been approved to treat locally advanced and metastatic cSCC. Its use is safe and effective and must be considered a first-line treatment in such cases [5,6].

In this context, a multidisciplinary therapeutic approach to cSCC of the scalp acquires substantial importance, especially in those cases of disease that are already advanced at the moment of clinical presentation, in which other therapies besides surgery may play a crucial role.

Based on our surgical experience and a literature review, we aimed to propose a therapeutic algorithm used in our multidisciplinary group for squamous cell carcinoma of the scalp. Our purpose was to give a further instrument for a more systematic approach to the reconstructive choice, in particular for locally advanced lesions, while also illustrating how surgical therapy intersects with other therapies, with a view to multidisciplinary disease management.

## 2. Materials and Methods

Our algorithm is based on a retrospective analysis of a series of 202 patients who underwent resection of a squamous cell carcinoma of the scalp and contextual reconstruction over an almost 7-year period, from January 2016 to August 2022, with 243 malignancies overall. The mean age of our population was 81.35 years (median 82), ranging between 57 and 99. The male-to-female ratio was 8.6:1 (Table 1).

There were no distinct exclusion criteria in patient selection. We retrospectively selected our cases by collecting all cases of squamous cell carcinoma of the scalp in the aforementioned period, starting from histological reports. We then found the personal and clinical data of the patients and finally imported all information to an Excel database.

**Figure 1:**
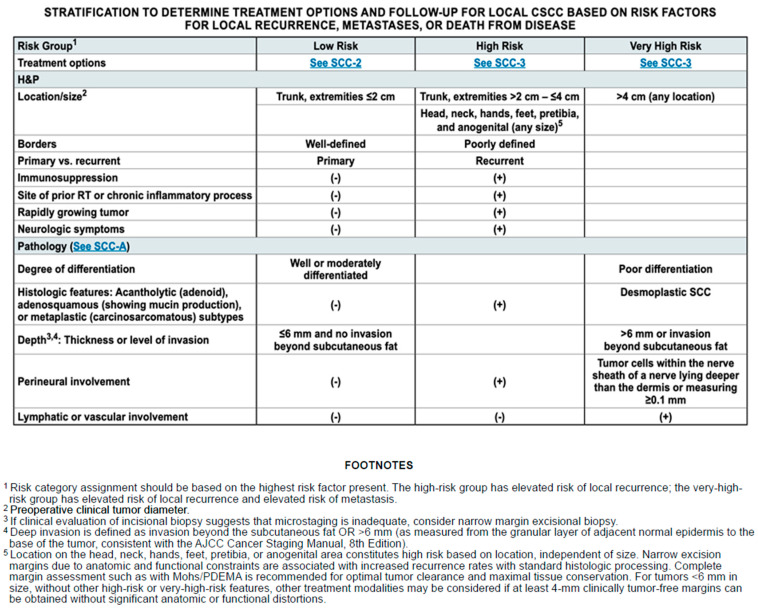
Risk stratification according to NCCN guidelines for local cSCC. Scalp cSCC falls into the high-risk or very-high-risk category based on clinical and pathological features of the tumor [7].

Among our case series, we distinguished between high-risk and very-high-risk lesions of the scalp, according to the SCC NCCN guidelines, based on the clinical and pathological features of the tumor [7] (Figure 1).

We then collected the surgical procedures for all patients, and the reconstructive choices were related to the extent and depth of malignancies and to the patient’s clinical status. We finally focused on the second group of lesions, classified as very-high-risk, and, in particular, on extensive and full-thickness defects, to evaluate the type of complex reconstruction performed.

At the end of data collection, our database included all the following personal, clinical, oncological, and surgical data: date of birth of the patient, age, gender, comorbidities (with particular attention to chronic or immunosuppressive disease and personal history of other oncologic pathologies or other previous skin cancers or recurrent lesions), clinical and pathological features of the malignancies, date of surgery, and surgical procedures with a description of reconstructive choices. The follow-up period was a minimum of one year.

## 3. Results

A total of 202 patients, 181 men and 21 women, with a mean age of 81.35 years (range 57–99), underwent 243 scalp reconstructions following malignant tumor resection.

The size of the analyzed tumors varied significantly, ranging from small lesions measuring less than a centimeter to malignancies that involved most of the scalp, with a major diameter of up to 17 cm. Among the 243 scalp squamous cell carcinomas assessed in our case series, 188 were classified as high-risk tumors, while 55 exhibited clinical and/or pathological features indicating a very-high-risk profile.

The vast majority of the tumor cases examined were primary excisions, with only 15 cases involving re-excisions due to recurrent or incompletely excised malignancies.

Among the reconstructions carried out, we report 76 primary closures for small defects, 115 reconstructions with skin grafting, 7 reconstructions with dermal substitutes in cases of periosteum infiltration, 33 local flaps, 1 locoregional flap (pedunculated trapezius), and 1 microsurgical free flap (latissimus dorsi) for resections up to the dura mater performed with the neurosurgical team. The remaining cases involved patients not eligible for surgery who, after histological confirmation, were sent to radiotherapy or immunotherapy with Cemiplimab. Given these findings, upon examining the tumor characteristics—specifically their extent and depth of infiltration—it becomes feasible to correlate them with the optimal approach for reconstruction and treatment. 

More specifically, we found that partial-thickness defects with a small diameter after malignancy resection, up to 3 cm maximum (the majority being <1 cm or from 1 to 2 cm diameter defects), benefited from primary closures due to the intrinsic elasticity of the skin.

**Figure 2:**
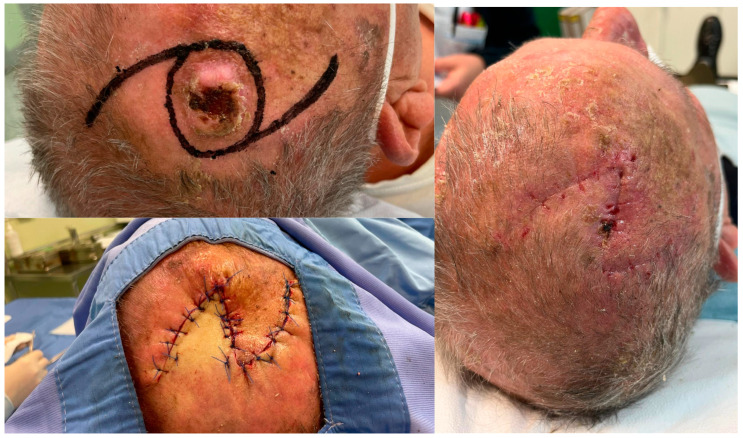
Example of an O-Z flap set up for reconstruction of a medium sized defect of the parietal region.

Local flaps set up from adjacent scalp skin were used for partial-thickness small–medium-sized defects, from 1 to 5 cm; the most frequently used were rotation flaps (hatchet flap), rotation–advancement flaps, and transposition flaps (like O-Z flaps) (Figure 2).

**Figure 3:**
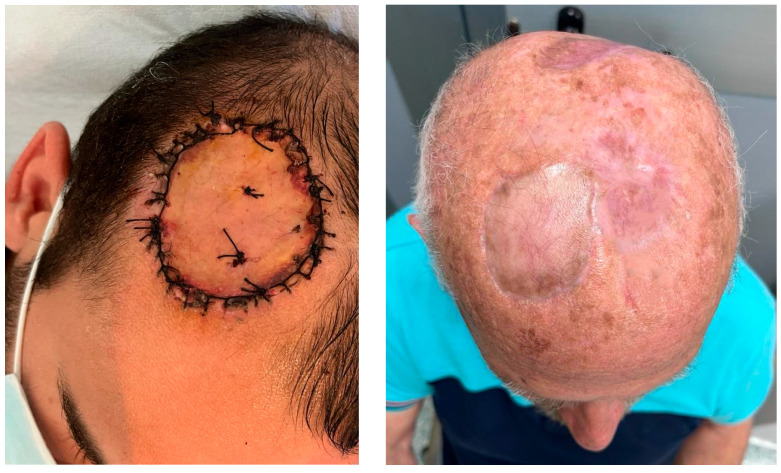
Skin grafts are usually used when aesthetics are not a concern and in the elderly population. However, for older and bald male patients, skin grafts represent a good option aesthetically, regardless of the size of the defect.

Skin grafting, to partial or full thickness, was used for partial-thickness medium–large-sized defects with a maximum diameter of 10 cm, with intact underlying periosteum (Figure 3); in just one case, there was an intraoperative finding of periosteum infiltration, so a small area of the periosteum was removed at the central portion of the lesion and a graft was immediately performed to remedy the loss of substance; after a histological confirmation of deep margin infiltration, the patient was referred to our Non-Melanoma Skin Cancers tumor board and candidated for immunotherapy.

**Figure 4:**
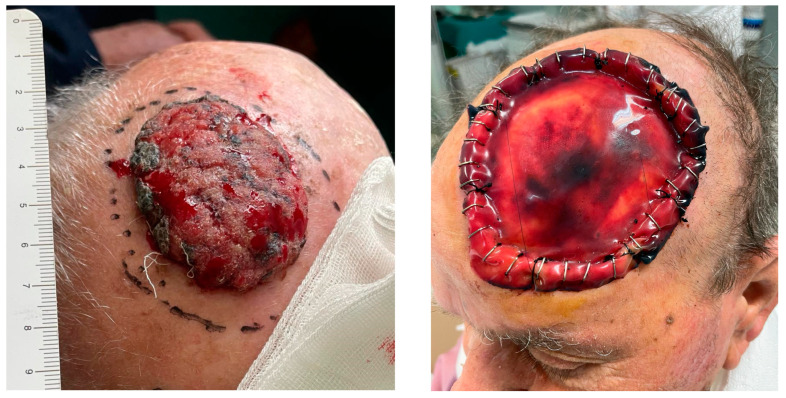
Clinically hypomobile extensive squamous cell carcinoma of the scalp. Considering the deep tissue infiltration documented during the intraoperative phase and the need for periosteal removal, we found the best reconstructive option to be a double-layer dermal matrix, followed by skin grafting after three weeks.

More complex surgical and therapeutic approaches were collected for more locally invasive lesions. In particular, in cases of partial or massive periosteum infiltration, the reconstructive choice fell on dermal substitutes (Figure 4) in a double layer with subsequent skin grafting after, on average, three weeks (five cases) or a single layer with contextual skin grafting (two cases). In all cases, the resection included the underlying periosteum, and in two of them, with intraoperative evidence of cranial bone infiltration, bony biopsies were sent for histological confirmation. For just three patients, the surgery was oncologically radical. For the other four, histology showed involvement of the deep margin of resection, so after a multidisciplinary assessment, the patients were sent to adjuvant therapies; in the first instance, radiotherapy was administered, but if this was not feasible because of a high risk of toxicity or because of prior irradiation, immunotherapy with Cemiplimab was proposed. 

One patient with a large squamous cell carcinoma of the scalp, located in the retroauricular region, measuring 6 × 5 cm and clinically hypomobile on underlying plans, underwent surgery with the support of the neurosurgical team. Lesion resection was performed with cranial bone full-thickness removal up to the dura mater, then a locoregional flap of pedunculated trapezius was set up for reconstruction; after four days, because of the flap suffering from venous congestion, the patient was returned to the operating room for flap removal and rescue reconstruction with the dermal substitute Integra^®^. The histological exam confirmed complete excision of the tumor, with a deep margin 1 cm clear. However, after about 2 weeks, the wound was still dehiscent due to failed engraftment of the dermal substitute, so it was removed. A new double-layer dermal regeneration template (Pelnac^®^ Gunze, Tokyo, Japan) was positioned together with a VAC dressing, set to 50 mmHg continuous negative pressure. After about 1 month, the dermal matrix appeared to be well engrafted on the dura mater and the residual bone, so the silicone layer was removed, and a neurosurgical team proceeded with superficial debridement on the dural surface and bone drilling in the occipital region up to the periosteum and in the mastoid region. The reconstructive team set up a temporoparietal transposition flap, based on the superficial temporal artery, to cover the front two-thirds of the defect including the dura, and they performed nuchal scalp advancement to cover the residual posterior loss of substance; therefore, a partial-thickness skin graft taken from the thigh was placed to cover the donor site of the flap. Finally, the remaining loss of substance in the anteroinferior region was left to heal by secondary intention and followed up with medication. This surgical procedure was definitive, no complications were reported, the patient healed completely, and no tumor relapse occurred within more than two years of follow-up.

**Figure 5:**
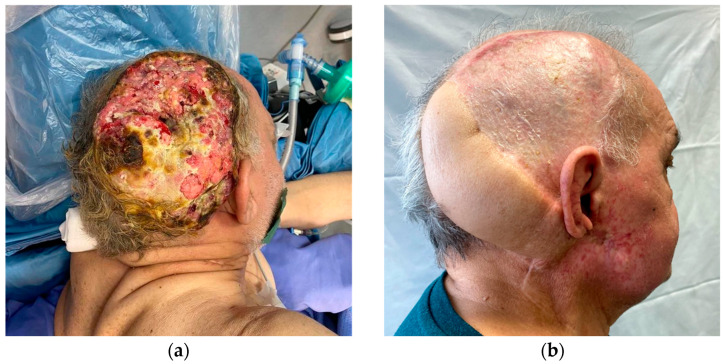
Clinical presentation of an aggressive ulcerated squamous cell carcinoma of the scalp in the parietomastoid area, undergoing resection hesitantly in a large full-thickness defect with removal of a large portion of the parietal bone (**a**). Reconstruction performed with a latissimus dorsi free muscular flap covered by a partial-thickness skin graft: appearance after two years of follow-up (**b**).

One patient presented with aggressive ulcerated squamous cell carcinoma of the scalp in the parietomastoid area, measuring 17 cm craniocaudally and 11 cm anteroposteriorly, with evidence of infiltration of the underlying bone structures in the contrast-enhanced magnetic resonance imaging. Combined resection and reconstruction surgery was planned with the neurosurgery colleagues: the neurosurgical team first removed the tumoral mass and performed right parietal craniotomy and mastoidectomy, the ENT colleagues performed a right laterocervical node clearance, and the plastic surgeons reconstructed the defect with a latissimus dorsi free muscular flap; vascular anastomoses were performed between the subscapular artery and the superior thyroid artery and between the subscapular vein and the external jugular vein; then, a partial-thickness skin graft was used to cover the muscular flap. Histology demonstrated the deep margin of surgical excision involvement, and residual neoplasm was shown in the retroauricular area in a postoperative CT scan. The patient was not considered suitable for adjuvant radiotherapy because of their severe depression and anxiety disorder, so an indication was given to start immunotherapy with Cemiplimab. After six months, a CT scan demonstrated reduced local recurrence thanks to immunotherapy. Still, one year after surgery, because of the clinical progression of the disease, radiation therapy was indicated with concomitant immunotherapy, with surprising progressive clinical and instrumental improvement. The combination of these two therapies led to down-staging of the local recurrence and surgical excision was then possible, with a definitive histology negative for disease. Today, after more than two years of follow-up, the patient is still free from disease [6] (Figure 5a,b).

Surgical treatment was radical and conclusive in the vast majority of cases. In 23 cases, adjuvant treatment was necessary, leading to either complete healing or at least good local disease control. We considered radiation therapy as an adjuvant treatment after surgery when surgical excision of the tumor was not complete, when margins were uncertain or clear but close (<1 mm), and when further surgery was not possible or was not accepted by the patient in the presence of tumors considered at high risk of local recurrence. Immunotherapy was considered for locally advanced cSCC, also in conjunction with radiotherapy.

Most of the patients under study presented with neoplasms susceptible to first-line surgical treatment. The remaining cases (10) involved patients not eligible for surgery because of the extent of the tumor and/or documented infiltration of deep layers or distant metastasis: first, incisional biopsies of the lesions were performed, and then, after histological confirmation, patients were referred to radiotherapy or immunotherapy with Cemiplimab.

Overall, 11 patients experienced local recurrence of the disease between 8 months and 4 years from the first resection. Two patients presented more than 1 recurrent lesion, with 13 recurrences overall. The percentage of recurrence among all the surgeries carried out was 5.35%. All of them underwent surgery again, and most of the recurrent lesions were reconstructed with skin grafts (two dermal substitutes, one local flap).

**Scheme 1:**
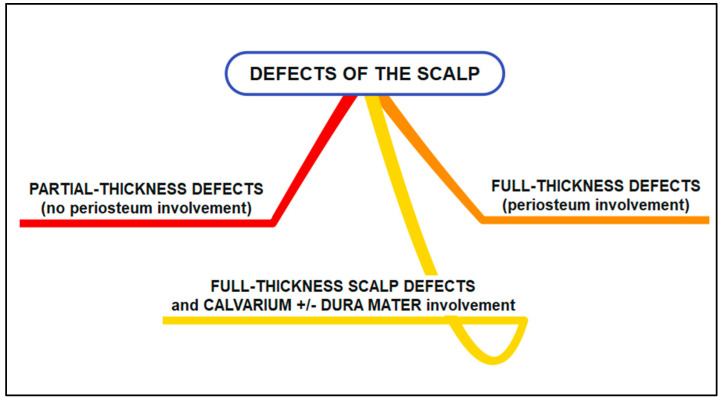
For a full comprehensive elaboration of the reconstructive algorithm, scalp defects were categorized according to depth into partial−thickness or full−thickness defects based on the periosteal involvement, and as complex defects involving cranial bone and inner structures.

**Scheme 2:**
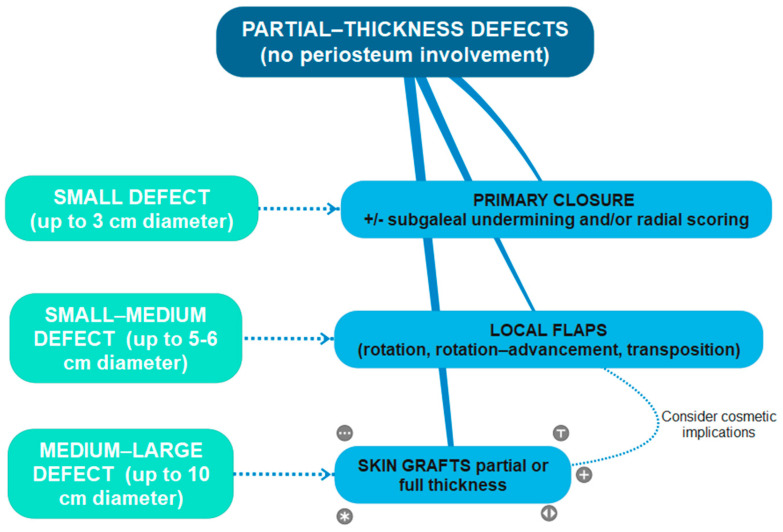
Reconstructive algorithm for partial-thickness defects, without periosteal involvement, according to depth and extent parameters.

**Scheme 3:**
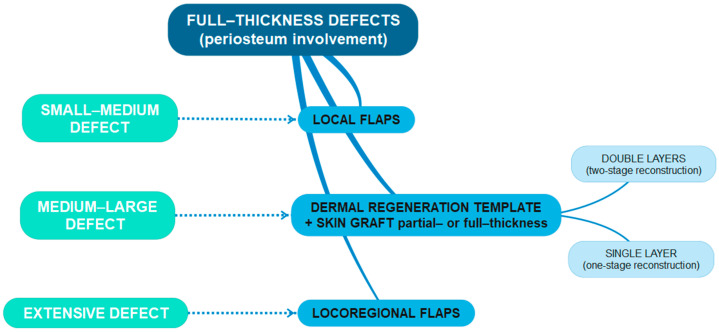
Reconstructive algorithm for full-thickness defects, with periosteal involvement, according to depth and extent parameters.

**Scheme 4:**
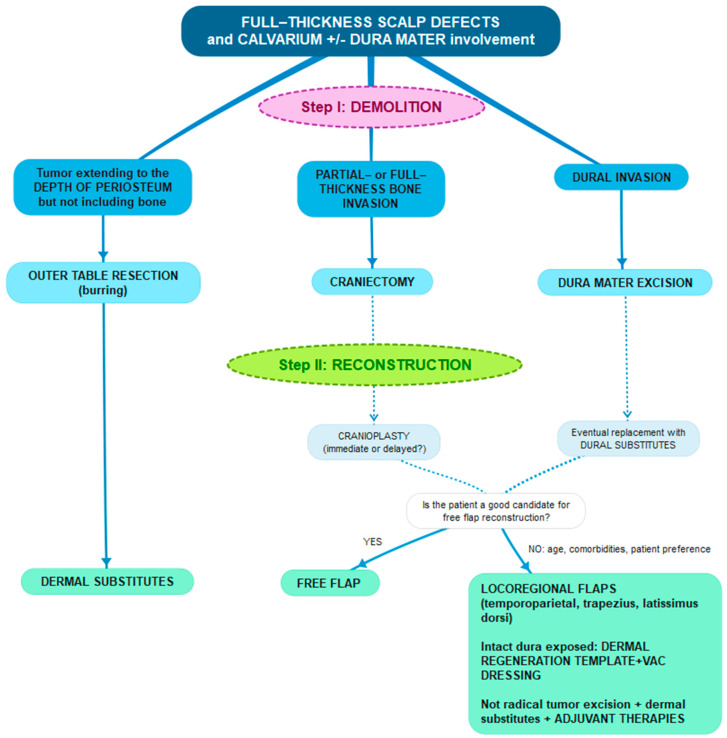
Algorithm of surgical management for complex full-thickness defects with calvarium and dura mater involvement.

**Scheme 5:**
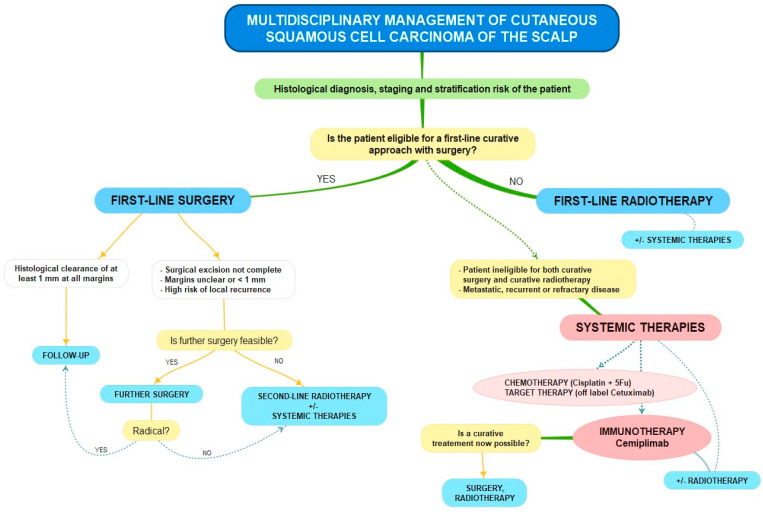
The comprehensive algorithm for an effective approach to scalp cSCC demonstrates how surgery intersects with other therapies, such as radiotherapy and immunotherapy, for multidisciplinary management of the disease.

An analysis of these results, together with a literature review on the methods proposed so far for the surgical and medical management of cSCC of the scalp, allowed us to develop a therapeutic algorithm for the overall management of this disease, which can be graphically represented as follows (Scheme 1, Scheme 2, Scheme 3, Scheme 4 and Scheme 5).

## 4. Discussion

Cutaneous squamous cell carcinoma is nowadays one of the most common malignancies that we encounter in our clinical practice, with a rapidly increasing incidence rate probably due to the progressively aging population and the greater attention to skin cancer screening [8]. 

The scalp is one of the main locations of cSCC, which presents with high-risk or very-high-risk features and can often show local invasion at the moment of clinical presentation. Therefore, since advanced cSCC of the scalp may impose significant risks in terms of impacts on quality of life, morbidity, and mortality, as well as increased costs for management, a systematic approach to treatment is mandatory [2,9].

The first aspect to consider in the management of cSCC of the scalp is surgery and proper reconstruction of the defects. When patients are eligible for surgery, radical resection represents the first-line therapy, improving prognostic outcomes and laying the foundation for following adjuvant therapies [10]. As the tumor grows in size and depth, with possible involvement of the bone and inner structures, cSCC scalp resection can result in complex wounds, which may represent a challenge regarding reconstruction. This has become a topic of multidisciplinary interest, as nowadays many surgical specialists are involved in performing scalp reconstructions.

Various reconstructive options have been described in the literature, with their advantages and disadvantages, possible complications, and related functional and aesthetic implications [11].

For partial-thickness defects without periosteum involvement (Scheme 2), we chose primary closure for the smallest defects up to 3 cm in diameter, with excellent aesthetic results since it makes use of adjacent hair-bearing tissue; as is well known, this method is typically limited by the low elasticity and pliability of scalp skin with the risk of excessive closure tension, which should be avoided to prevent wound dehiscence and prevent hair follicles from suffering. We partially overcame this problem by wide tissue undermining in the subgaleal layer and, if necessary, radial scoring [3,11]. Tissue expanders might be another option but are not usually used in the oncologic setting, nor were they considered in our patients’ surgical management, because of the delay they would cause in curative resection of the tumor [12].

In our experience, skin grafting is a reliable method of reconstruction for partial-thickness medium–large-sized defects up to 10 cm in diameter, as it is quick and easy to set up with minimum donor site morbidity, especially for full-thickness skin grafts. The bed for grafting should rely on an available nutrient blood supply, possibly represented by intact periosteum; therefore, when the periosteum after tumor resection was not preserved for oncological safety reasons, we preferred to rely on other reconstructive options, primarily the use of dermal substitutes. Alternative solutions reported in the literature are represented by drilling of the outer table of the calvarium to expose the diploic space and stimulate granulation tissue [13] or, less frequently, the setting up of pericranial flaps [14] or subgaleal fascia flaps [15]. We preferred dermal substitutes with subsequent skin grafting to skin grafts alone in cases of expected adjuvant therapies because, as known, skin grafts often heal into unstable scars; therefore, this approach is not recommended if the patient is possibly a candidate for postoperative radiotherapy [3,11,16]. 

Since skin grafts cannot provide hair-bearing scalp and often result in unaesthetic scars because they offer a poor match with the color, structure, and thickness of the native scalp, they are usually used when aesthetics are not a concern and in the elderly population. Instead, local flaps from the adjacent regions show the best color and tissue quality match and the ability to bear hair. In our experience, however, for elderly and bald male patients, skin grafts represent a good option aesthetically, and, regardless of the size of the defect, we preferred them to local flaps. On the contrary, local flaps are best suited for younger patients, women, and, in general, whenever it is necessary to replace hair-bearing scalp, particularly safeguarding the temporal and frontal hairline. For these reasons, our opinion in choosing the most appropriate surgery for partial-thickness medium and medium–large scalp defects is to rely not only on tumor and defect clinical parameters but also on the aesthetic implications for the individual patient, by customizing the reconstructive choice.

Regarding local flaps, we used them mainly for small–medium-sized partial-thickness defects, and the most reliable ones in our experience were single- or double-rotation flaps like the classic hatchet flap or O-Z flap. Their use in our series was sometimes limited by the high-risk clinical features of the malignancy and by the necessity of histopathologic confirmation of tumor eradication [10], so when the clinical margins of the lesion were unclear and poorly defined, we often preferred skin graft reconstruction, which allows for easier reoperation if enlargement is necessary and better control of any recurrence during the follow-up period.

Moreover, wide scalp flaps are best suited in cases of calvarium exposure because they provide well-vascularized tissue. The best results are reached with large flaps (four to six times wider than the original defect) with rotation or rotation–advancement movement, because curvilinear incisions are more compliant with the cranium’s natural convexity [3,4,17].

When we had full-thickness defects with bone devoid of periosteum (Scheme 3), dermal regeneration templates proved to be a safe and reliable reconstructive tool for scalp defects. This technique allowed us to overcome reconstructive limits due to defect size and location or a lack of local donor sites because of previous radiation or a history of multiple and recurrent scalp tumors. The advantages are represented by shorter operating times and faster patient recovery, with a low complication rate. They offer a more stable tissue covering than skin grafts and are supple and uniform in texture, which can provide support following radiotherapy [18,19]. Moreover, the use of dermal substitutes in our experience represented an excellent functional and aesthetic option with lower morbidity and mortality, even in the highly elderly population [20]. For this reason, we preferred artificial dermis as a valid and reliable alternative to autologous tissue, especially in older patients with severe pathologies, recurrent skin cancers, and the necessity of adjuvant or neoadjuvant radiotherapy [21]. In our experience, we often used porcine- and bovine-derived acellular dermal matrix (Integra^®^-Integra Lifesciences Corporation, Princeton, NJ, USA- and Pelnac^®^-Gunze, Tokyo, Japan-products), performing two-stage or one-stage (if a single-layer device was used) procedures, with favorable functional and aesthetic outcomes. In addition to the above advantages, dermal substitutes helped us in critical situations where the patient’s clinical conditions did not allow for more aggressive surgical procedures, acting as a “bridge” cover while waiting for histological results and further therapeutic indications.

The most critical issues are posed by squamous cell carcinoma involving the full scalp thickness with bone and even dura mater invasion (Scheme 4). Since there is no universal protocol for these complex patients, their therapeutic management must be conducted by trained, experienced, and multidisciplinary teams. Numerous recommendations are reported in the literature for scalp reconstruction after local aggressive squamous cell carcinoma resection, while indications for the demolition phase regarding whether to proceed with the removal of the outer table or with a full craniectomy are poor.

Given that full-thickness craniectomy is indicated when cortical bone invasion or an intracranial tumor extension is present, the question may be whether, in cases of scalp SCC extending to the depth of the periosteum but not including bone, the more aggressive craniectomy is superior to a more conservative approach. Therefore, a crucial point in our opinion may be how to behave when preoperative imaging excludes bone involvement but we clinically suspect a certain degree of infiltration because of, for example, hypomobility of the lesion. Our multidisciplinary algorithm requires that in patients with known or suspected periosteal invasion or a positive deep margin after histology, the neurosurgical team proceeds to resect the outer cortex of the calvarium by burring the outer table down to the diploe. At the same time, full-thickness resection is performed on patients with evidence of extensive calvarial invasion on preoperative imaging. For tumors involving the periosteum but not including bone, we preferred outer table burring to craniectomy. This method limits the risks and complications related to an aggressive procedure such as craniectomy, ensuring optimal oncologic outcomes. The main disadvantage of this procedure is that the bone fragments produced by burring are hardly subject to pathologic analysis. Other methods of outer table resection are possible, like outer table splitting. This approach allows for pathological examination of the outer table, but this is a more complex surgical procedure, burdened by an increased risk of intracranial penetration and dural tear [22,23]. A recent retrospective review by Garrison and colleagues [24] argued that in such settings with exclusive involvement of the periosteum, soft tissue excision with resection of the outer table only is an excellent option, offering a low rate of local recurrence and high disease-free survival, while mitigating the significant morbidity and mortality associated with the alternative option of a full-thickness craniectomy and subsequent free flap [24,25]. In their practice, the scalp was excised with at least 1 cm margins and down to the calvarium, then sent for frozen pathology to assess the margins. If invasion of the deep margin was found, the periosteum was considered positive. They then proceeded with meticulously burring the outer table of the cranium until reaching the vascular diploic space, then reconstructing with a local flap or dermal regenerative template followed by a split-thickness skin graft. 

Regarding reconstruction after this procedure, our preference falls on dermal substitutes because, having no possibility of histological control of the bone removed by burring, this tool allows for more reliable monitoring of any local recurrence during the follow-up period and easier eventual reoperation as compared to flaps; these advantages are also offered by skin grafts, which may eventually survive thanks to vascularization of the exposed diploic space. However, a dermal matrix offers a more stable tissue covering than skin grafts and easier engraftment onto exposed structures. Although good results are reported in the literature regarding local control of the disease and disease-free survival with this conservative approach, further studies are necessary with broader cases to demonstrate whether it is comparable to craniectomy regarding oncological safety [26,27]. We believe that the choice must also be guided by patient status, since elderly patients with many comorbidities can safely benefit from sparing time and surgical risks.

Instead, when cortical bone and dura invasion were present, to ensure radical tumor removal, a craniectomy and dura mater excision were required.

Traditionally, various methods have been described for bony tissue removal, each with its own drawbacks, including thermal and physical damage to the bone or surrounding structures, as well as limited precision. The limitations associated with traditional bone removal methods have led to the development of novel tools, such as piezoelectric saws [28]. A critical precautionary step during osteotomy is to limit the amount of heat generated during sawing. While some authors have suggested using a multiple-drill-hole technique to mitigate thermal damage, this approach does not completely prevent bone heating; moreover, final cutting with a scalpel does not consistently guarantee accurate incisions or prevent undesired fractures. On the other hand, utilizing the piezoelectric effect allows for a more controlled incision with minimal temperature fluctuations and reduced risk of cellular damage to the bone. Piezoelectric saws use ultrasonic vibrations to precisely cut bone while minimizing damage to surrounding tissues. This technique also appears to lower the levels of certain pro-inflammatory cytokines in the bone, potentially encouraging new bone formation. Additionally, the piezoelectric saws’ precision significantly lessens the need for extensive retraction and stretching of soft tissues, minimizing trauma and enhancing the accuracy of the procedure. In summary, by reducing the risk of thermal and physical damage, piezoelectric saws help improve surgical outcomes and patient recovery [29]. Craniectomy and dura mater excision lead to the creation of a complex wound that exposes the patient to the risk of cerebrospinal fluid leak and infection; after these procedures, there is no agreement about the indication of cranioplasty for cerebral protection and maintaining the contour of the calvarium [30]. Performing a cranioplasty in the immediate reconstruction remains a controversial point because, as reported by several studies [31,32], the risk of postoperative infections—which, in most cases, require the removal of the implant—is considerably higher; for these reasons, our team’s preference is not to perform it. Eventually, we can reserve the possibility to proceed with a delayed skull reconstruction, which results in a lower complication rate [33].

As is well known, cranial bone reconstruction can be performed alternatively through autologous reconstruction techniques or using alloplastic materials. Autologous reconstruction encompasses the utilization of vascularized or non-vascularized bone. In cases where the affected area of the skull is not so extensive, split calvarial grafts can be sourced from other regions of the calvarium itself, particularly the parietal bone, which tends to be the thickest. For more substantial defects, ribs are a more suitable option, with the seventh and eighth ribs being the most frequently utilized. To preserve chest wall stability, it is advisable not to harvest more than two or three ribs, and to prevent pleural damage, a subperiosteal harvesting approach is recommended. When dealing with wounds that have been exposed to radiation or infection, vascularized bone flaps are highly beneficial. In such cases, the serratus anterior flap or a chimeric latissimus dorsi–serratus anterior flap, including a portion of vascularized cartilage or scapular bone, can be considered. Alternatively, a chimeric scapular or parascapular flap with the inclusion of a segment of the scapula may also be suitable. In more complex scenarios, the free fibula flap, which is renowned for its versatility in the design and shape of the bone component, has also been described [34,35].

Reconstruction with alloplastic materials (titanium mesh, polymethyl methacrylate, porous polyethylene, calcium phosphate cement, polyetheretherketone, and so on) is more commonly performed, as these materials are widely available and have minimal donor site morbidity; however, they are much more prone to infection than autologous bone grafts, which may eventually preclude their use in infected or previously irradiated areas. The ideal alloplastic implant should be compatible with the surrounding bone and soft tissues, resistant to infections, strong, easily shaped, and stable over time [36,37].

Regardless of cranioplasty, for extensive and full-thickness scalp and calvarium defects, we believe that reconstruction with microsurgical free flaps provides the most safe and reliable repair method, offering well-vascularized tissues that can support adjuvant treatments. Free flaps have become the best choice, especially in complex settings like prior tissue irradiation, neurocranial structure exposure, cranial bone reconstruction with alloplastic material, a history of chronic infection, or previous reconstruction failure [3]. They provide a large amount of well-vascularized tissue that better contours to the calvaria’s convexity and supports eventual adjuvant radiotherapy with minimal soft-tissue complications. The free latissimus dorsi muscle transfer is nowadays considered the mainstay to cover complex scalp defects and tolerate radiation [30]. For our patient with extensive SCC of the parietomastoid area, we chose a latissimus dorsi free flap, benefiting from its constant and reliable pedicle and its capability to adhere better to the underlying surface without bulkiness, offering an excellent aesthetic appearance when covered with a split-thickness graft. Despite its known limitations, such as donor-site morbidity, this flap remains one of the most commonly used reconstructive solutions when a large amount of tissue is needed for subtotal or total scalp defects. Besides the ability to raise a composite flap with multiple muscular and non-muscular components, according to the complexity of the defect, its greatest advantage is probably related to the excellent cosmetic results it provides; this is because the muscle atrophies over time, allowing the flap thickness to approach the natural thickness of the scalp [38].

Many flaps have been described in the literature for use in microsurgical scalp reconstruction, including muscle flaps covered by skin grafts and fasciocutaneous flaps [39,40,41]. Dr. Ehrl and colleagues, in a recent study, compared the characteristics of three standard workhorse-free flaps: the anterolateral thigh flap, the latissimus dorsi muscle flap, and the gracilis muscle flap [3]. Compared with the other two, a significantly shorter vascular pedicle often limits the gracilis muscle flap; however, it provides optimal adhesion to the underlying surface, and it offers great variability in shape, as it can be used to fill deep defects or can be flattened out to cover large areas [38]. The anterolateral thigh flap (ALT) is rapidly gaining popularity in scalp free flap reconstruction due to its numerous advantages: besides its low donor site morbidity, it can rely on a long vascular pedicle and great versatility in design and composition. Unlike the latissimus dorsi flap, the ALT allows for simultaneous scalp resection and flap harvest, facilitating a two-team approach that substantially shortens operative times; it causes minimal functional morbidity, and it can be harvested as an adipocutaneous, fasciocutaneous, or chimeric flap, eliminating the need for additional skin graft coverage. Still, the subcutaneous tissue’s thickness represents this flap’s main disadvantage, often requiring secondary debulking procedures; providing significant tissue bulk, the ALT flap may not adequately cover total scalp defects; it is thicker and less pliable compared to a latissimus dorsi flap; and furthermore, while the pedicle length of ALT flaps is comparable to that of LD flaps, the pedicle anatomy is more variable, which can make its harvest more challenging. The latissimus dorsi muscle flap has the highest reported donor site morbidity but represents an excellent option for larger or whole-scalp defects because the muscle is flat and wide, so it is the one that better covers and contours around the cranium [3].

However other alternatives can be considered, each with its own advantages and disadvantages. The radial forearm flap is not commonly used for scalp reconstruction, as it may not provide sufficient soft tissue coverage for large scalp defects compared to alternative flaps. Nevertheless, it has been demonstrated to be effective for smaller defects, typically those less than 7 cm wide. One of its advantages is its reliable and very long pedicle with good-caliber vessels, which simplifies dissection and harvest; additionally, it allows for a two-team concurrent resection and harvest, leading to shorter operating room times [38].

The rectus abdominis flap, despite a decrease in its usage over time, was historically popular for scalp reconstruction, thanks to its reliable anatomy allowing for ease of harvest. Above all, it provides significant tissue for total scalp defect coverage, and the donor site often allows for primary closure with satisfactory cosmesis. However, its major disadvantage is the increased risk of hernia formation. Another flap option for subtotal and total scalp reconstruction is the omental free flap. Covered by a skin graft, it can offer a large amount of thin, pliable soft tissue with a very long pedicle. However, its use requires a laparotomy, which adds complexity to the surgical procedure [38].

Although several studies have demonstrated free flaps’ safety and efficacy in the elderly population [42,43], the associated comorbidities frequently presented by elderly patients—with cardiac disorders first among them—represent another significant challenge since they limit anesthetic tolerance and the use of distant flaps [44]. In these settings, locoregional flaps can be used for wide and complex defects when a large amount of vascularized tissue is needed but the patient is not a good candidate for free tissue transfer. For our patient, the reconstructive choice fell on the trapezius pedunculated flap because the tumor was located in the retroauricular region and was not too extensive, but a full-thickness resection up to the dura was necessary. The most commonly described regional flap for scalp full-thickness defect reconstruction includes the temporoparietal fascia for anterior and temporal areas, usually combined with a split-thickness skin graft to protect deep layers [10,45], and a pedunculated trapezius flap and latissimus dorsi musculocutaneous flap for posterior areas of the scalp [46,47]. Today, their indications are limited by the affirmation of free-tissue transfer and by the defect’s extension and location. Despite the length of their dominant vascular pedicle, these flaps cannot reach every region of the scalp, and overstretching the pedicle often leads to ischemia at the distal part of the flap [11,48].

Large full-thickness scalp and calvarial defects with exposed dura, when the patient is unfit for free flap reconstruction, may also be reconstructed with a dermal regeneration template together with a VAC dressing, achieving durable and reliable coverage [49]. This method can also be applied to chronically infected, non-healing scalp wounds with exposed bone as a bridge covering a definitive free flap reconstruction, when allowed by the patient’s medical condition [49,50]. This is not an approach we use systematically, especially in the context of neoplasm treatment. Still, it revealed some benefits in a previously described case in which we used a dermal matrix with a VAC dressing after a previously failed reconstruction, using this method as a temporary covering to prepare tissues for definitive reconstruction.

Generally, each surgical approach must be tailored to the patient’s clinical conditions and preferences [16]. For example, in our series, patients with a long-standing neglected scalp squamous cell carcinoma with suspected or documented bone and inner structure infiltration, who probably had required large full-thickness local excision and complex reconstruction, instead received a more conservative approach when limited by age, cognitive impairment, anesthesiological contraindication for underlying disorders, or difficulties in home care. Most of all, this category of patient needs a careful upstream case assessment in a multidisciplinary context to identify the best option for the patient. According to a multidisciplinary decision, in these cases we often performed less destructive resections and less demanding reconstructions, like with skin grafts or dermal substitutes, followed by adjuvant therapies, or alternative first-line radiotherapy or immunotherapy after histological diagnosis. 

When facing an R1 resection, where there is no residual macroscopic tumor but, microscopically, the surgical margins still demonstrate the presence of tumor cells, we proceed whenever possible with the enlargement of the previous excision, especially targeting the involved margin(s). In these cases, as in the presence of unclear and poorly defined clinical margins of the lesion and for tumors prone to local recurrence, we often preferred skin graft reconstruction, which allows for easier eventual reoperation and better control of any recurrence during the follow-up period.

If the required enlargement involves the removal of the periosteum and possibly part of the bone, dermal substitutes have proven to be a reliable option. They provide a stable tissue covering and easier engraftment onto exposed structures compared to skin grafts.

Alternatively, another approach could be to delay further reconstruction after enlargement while awaiting histological confirmation of radical tumor removal. In this scenario, the patient would be managed with dressings until histological confirmation is obtained. Once confirmed, definitive reconstruction can be safely performed, including the use of local flaps if necessary. This approach ensures that the reconstruction is actually based on confirmed tumor clearance, minimizing the risk of recurrence.

Most problems may arise in locally advanced and recurrent cases of squamous cell carcinoma of the scalp, with the main risk being wasting resources and unnecessarily lengthening the treatment time if a systematic approach to the disease needs to be set up from the beginning. The management of complex cases often required us to combine surgery with other therapies, like radiation therapy, chemotherapy, and immunotherapy, and to optimize multidisciplinary care. Our multidisciplinary tumor board dedicated to Non-Melanoma Skin Cancers (NMSC) is an important resource when a clear treatment path is not defined, particularly if many risk features are present and nonsurgical treatments are necessary.

According to the literature and guidelines, in our multidisciplinary decision algorithm, it is always essential at first to obtain a histopathologic diagnosis of the lesion to identify the necessary parameters, together with the patient’s clinical status, for risk stratification (Scheme 5). Nodal involvement is a critical prognostic factor, given that regional nodal involvement significantly increases the risk of recurrence and mortality, so early detection is mandatory. Furthermore, nodal metastasis often coincides with other adverse histopathologic findings such as lymphovascular invasion, poor differentiation, and perineural invasion [7]. For patients presenting with palpable lymphadenopathy or suspicious nodes identified via imaging studies, confirmatory tests such as ultrasound-guided fine-needle aspiration (FNA) or core biopsy of the involved lymph nodes are indicated, since the early recognition of nodal disease has a good impact on prognosis and may increase survival [51,52,53]. A positive result from fine-needle aspiration or biopsy usually indicates the need for lymphadenectomy of the associated nodal basin, with or without adjuvant radiation therapy depending on the specific circumstances.

The NCCN panel recommends discussing and considering sentinel lymph node biopsy for cases of very-high-risk cutaneous squamous cell carcinoma that are recurrent or with multiple risk factors, when local lymph nodes appear normal under ultrasound and demonstrate a normal examination of the draining nodal basin. While sentinel lymph node biopsy (SLNB) may provide prognostic value, it remains uncertain as to whether performing SLNB followed by the completion of lymph node dissection or adjuvant radiotherapy ultimately improves patient outcomes. 

Based on the suspected extent and diffusion of the squamous cell neoplasm, further imaging studies should be performed to complete risk stratification and staging [7].

After appropriate diagnosis and staging, we discuss whether patients with advanced cSCC are eligible for a curative approach with surgery, radiotherapy, or both. It is advisable to choose surgical excision as the first line of treatment whenever possible, ensuring at least a 1 mm histological clearance at all margins by including sufficient peripheral and deep tissues [54]. Still, in our experience, radiotherapy is a tissue-preserving curative method that can sometimes achieve better aesthetic and functional outcomes [26]. For this reason, when surgery is not feasible or would result in unacceptable functional or aesthetic morbidity, radiotherapy can be used as a first-line and even definitive treatment. We consider radiation therapy as a second-line treatment or as an adjuvant treatment after surgery when surgical excision of the tumor is not complete, when margins are uncertain or clear but close (<1 mm), and when further surgery is not possible or is not accepted by the patient in the presence of tumors considered at high risk of local recurrence (perineural invasion within a nerve >0.1 mm in diameter, recurrent disease, immunocompromised patients); in addition, radiotherapy could also be offered as a form of palliative care [54].

Patients with high-risk locally advanced or metastatic cSCC of the scalp face poor outcomes and few treatment options, as they are often ineligible for both curative surgery and curative radiotherapy. We consider such patients likely to be treated with systemic therapies and to be those with recurrent and refractory disease. The systemic treatments described for cSCC include chemotherapy, targeted therapy, and, more recently, immunotherapy [5,53,55]. The chemotherapy most often used to treat advanced cSSC involves cisplatin and 5-fluorouracil in combination [7]. Targeted therapy is an option since several studies have shown that up to 80% of cSCC tumors and 100% of metastatic cSCC tumors express epidermal growth factor receptors (EGFRs) [56]. The monoclonal antibody Cetuximab, preventing the ligand-induced activation of EGFR, has been used off-label as a systemic treatment option for patients with advanced cSCC [57]. However, the therapies mentioned above offer modest benefits, while adverse events are frequent and potentially severe.

The development of our understanding of immune checkpoints has led to the application of immunotherapy in several oncologic settings with profound and durable responses: anti-PD-1/PD-L1 drugs act by blocking the signaling between the PD-1 (programmed cell death-1) receptor and its ligand PD-L1, thus allowing for an antitumor response from the immune system. Immunotherapy has become popular for locally advanced or metastatic cSCC, often used in conjunction with radiotherapy [58]. The first drug that Health Canada and the U.S. Food and Drug Administration specifically approved for advanced cSCC treatment was Cemiplimab [59]. Cemiplimab is a monoclonal antibody that belongs to the class of immune checkpoint inhibitors. Specifically, it acts as an anti-PD-1 systemic immunotherapy, showing substantial antitumor activity in patients with metastatic and locally advanced cutaneous squamous cell carcinoma. We used Cemiplimab as a rescue therapy or palliative therapy when the patient was not a candidate for curative surgery or radiotherapy, with excellent results, and we also assessed the use of Cemiplimab in synergy with radiotherapy. In our patient with a crucial local disease recurrence [6], this therapeutic association allowed us to contain and understage the disease, leading the patient to a stage that was finally amenable to definitive curative treatment with surgery. 

Because of this experience, although Cemiplimab’s efficacy in the neoadjuvant setting remains unclear and this drug has not been approved for such use so far, we believe that immunotherapy in advanced cases, like for unresectable or metastatic disease, is a valuable treatment option to reduce the tumor mass and make surgery possible. Only a few recent studies have aimed to prove that in selected patients with locally advanced and aggressive cSCC, Cemiplimab may provide durable, complete pathological responses. Still, more evidence on more cases is needed to promote the use of this drug as a neoadjuvant therapy [60,61]. The evidence so far suggests that immunotherapy could be a promising option of care in the future, given its tolerability and efficacy [62]. Further studies are necessary to explore the potential application of this agent not only in adjuvant but also in curative settings, as well as to establish the best management for tumors that do not respond or relapse after anti-PD-1 immunotherapy and for immunosuppressed patients [5].

## 5. Conclusions

Today, our algorithm, based on an extensive patient case series, is a valuable tool used by our multidisciplinary team as a further guide for scalp cSCC therapeutic management. The development of this algorithm led us to reflect on some key points regarding cSCC treatment.

The reconstructive aspect is fundamental, even in the management of simpler defects. In addition to the characteristics of the extent and depth of the tumor, aesthetic and functional implications for the individual patient must always be considered; moreover, among the different reconstructive options, the one that allows better local control of the disease and eventual recurrence, depending on the patient’s risk level, may be preferred. In other words, the reconstructive choice must always be customized to the individual clinical situation.

Scalp cSCC with periosteal or calvarial involvement offers the most significant management challenge; for locally advanced cSCCs, more conservative surgical treatment methods can be considered, ensuring reasonable local disease control while exposing the patient to lower operational risks.

Managing squamous cell carcinoma of the scalp in locally advanced or recurrent cases requires a systematic approach to the disease, which combines surgery with systemic therapies. The decision-making algorithm implemented by the multidisciplinary team has improved outcomes for our patients. In fact, compared to the past, we have more therapeutic possibilities today than just radiotherapy or chemotherapy, which can be possibly used in combination with each other, even off-label. Thanks to systemic therapies, patients with advanced disease may be brought to a lower stage of disease and made eligible for curative treatments.

## Figures and Tables

**Table 1:** Demographic data of all patients assessed.

Number of patients	202
Male (%)	89.6
Female (%)	10.4
M/F ratio	8.6:1
Mean age	81.35
Median age	82
Age range	57–99
Total SCCs operated	243

## Data Availability

The data used and/or analyzed during the current study are available from the corresponding author upon reasonable request.
